# Role of IVUS in the rectification of angiographically judged ramus intermedius and its clinical significance

**DOI:** 10.1186/s12872-021-02034-1

**Published:** 2021-04-30

**Authors:** Xue Gong, Zheyong Huang, Zhonghan Sun, Qibing Wang, Juying Qian, Lei Ge, Junbo Ge

**Affiliations:** 1 Department of Cardiology, Deltahealth Hospital, Shanghai, 201702 People’s Republic of China; 2 Department of Cardiology, Shanghai Institute of Cardiovascular Disease, Zhongshan Hospital, Fudan University, 180 Fenglin Road, Shanghai, 200032 People’s Republic of China; 3 Human Phenome Institute, Fudan University, Shanghai, 200438 People’s Republic of China

**Keywords:** Ramus intermedius, Intravascular ultrasonography, Angiography, Coronary, Percutaneous coronary intervention

## Abstract

**Background:**

Due to the technical limitations of coronary artery angiography (CAG), ramus intermedius (RI) is sometimes difficult to distinguish from a high-origin obtuse marginal branch or a high-origin diagonal branch. This study sought to investigate the role of intravascular ultrasonography (IVUS) in the rectification of angiographically judged RI.

**Methods:**

This study retrospectively analyzed 165 patients who were reported to have an RI based on CAG and underwent IVUS implementation from 02/01/2009 to 31/12/2019 in Zhongshan Hospital, Fudan University. Taking IVUS as the gold standard, we calculated the accuracy of RI identification by CAG and evaluated the impact of RI on revascularization strategy.

**Results:**

Among the 165 patients, 89 patients (54%) were demonstrated to have an RI on IVUS (IVUS-RI), 32 patients (19%) were identified to have a high-origin diagonal branch on IVUS (IVUS-h-D), and 44 patients (27%) had an actual high-origin obtuse marginal artery on IVUS (IVUS-h-OM). Among 84 patients who underwent one-stent crossover stenting because of left main furcation lesions (48 patients in the IVUS-RI group, 12 patients in the IVUS-h-D group, and 24 in the IVUS-h-OM group), 14.6% of patients in the IVUS-RI group, 33.3% in the IVUS-h-D group and 0% in the IVUS-h-OM group had CAG-RI compromise (*P* = 0.02), which was defined as severe stenosis of the RI ostium (> 75%) or significant RI flow impairment (TIMI < 3).

**Conclusions:**

Only 54% of CAG-RIs were confirmed by IVUS, which indicates the necessity of preintervention IVUS to distinguish real RIs from other branches in LM furcation lesions.

## Background

The ramus intermedius (RI) is a variant coronary artery resulting from bifurcation of the left main coronary artery (LMCA) [1]. Generally, it is diagnosed on autopsy [2]. Recently, RI has increasingly been observed by computed tomography angiography (CTA) of the coronary artery [3–5]. The occurrence rate is ~ 20% (range 15–31%) of the population depending upon the series [3, 4].

Anatomically, RI is different from a high-origin obtuse marginal artery (h-OM) or a high-origin diagonal branch (h-D). Functionally, it is as important as these prominent early branches because it has a similar course and perfusion region to h-OM or h-D [1]. Once RI is occluded, patients can also have symptoms of chest pain, increased troponin levels and related electrocardiographic changes [6]. However, little attention has been paid to RI branch.

Percutaneous coronary intervention (PCI) with stent implantation has become a viable alternative to coronary artery bypass grafting (CABG) in patients with significant LM or LM bifurcation lesions [7]. However, PCI procedures for LM bifurcation lesions remain technically challenging [8–10]. The existence of RI changes an LM bifurcation lesion into a trifurcation lesion and changes the furcation angle [11], which makes PCI procedures more complicated. Therefore, it is important to distinguish RI from other prominent early branches.

Standard angiographic projections of coronary artery angiography (CAG) are often associated with vessel foreshortening and anatomical overlap [12]. This technique has a limited ability to capture the exact anatomy of the carina of the furcation or the ostial side branch (SB) [13]. Hence, it is reasonable to presume that many RIs reported on the basis of CAG do not truly originate from the furcation point. In contrast, intravascular ultrasonography (IVUS) is an accurate tomographic technique that is not affected by viewing angles; thus, it may overcome these shortcomings and might provide more valuable anatomical information than CAG [14].

The purpose of this study was to illustrate (1) the accuracy of CAG-reported RI using IVUS as the gold standard and (2) the impact of RI on revascularization strategy for the LM furcation.

## Methods

### Study population

Between January 2009 and December 2019, consecutive patients who had CAG-reported RI and underwent IVUS at Zhongshan Hospital, Fudan University, were enrolled. Patients were excluded in the event of suboptimal IVUS image quality or right coronary artery withdrawal. The study protocol was reviewed and approved by the Ethics Committee of Zhongshan Hospital, Fudan University. All patients provided written informed consent for the use of their data.

### Procedures and data collection

All CAG procedures were performed using standard coronary angiography projections. The standard fluoroscopic views included right anterior oblique (RAO) 30°, RAO 30°/cranial (CRA) 30°, RAO 30°/caudal (CAU) 30°, left anterior oblique (LAO) 45°/CAU 30° (“spider” view), LAO 30°/CRA 30° and CRA 30° for the left coronary artery, as well as LAO 45°, LAO 20°/CRA 20°, and RAO 30° for the right coronary artery. The “spider” view is often used to analyze the LMCA furcation and ostium of the left anterior descending artery (LAD) and left circumflex artery (LCX). If a SB can be seen coming out of the carina of LMCA furcation in the “spider” view, it will be reported as a RI by the cardiologists. PCI was performed according to the 2018 ESC/EACTS Guidelines on myocardial revascularization [15]. RI compromise was defined as severe stenosis of the RI ostium (> 75%) or significant RI flow impairment (TIMI < 3) [9, 16]. Each interventional cardiologist was responsible for the decision to employ a single or double stenting strategy for the treatment of LM furcation lesions. Demographic characteristics and clinical data were obtained from electronic medical record review.

### IVUS imaging acquisition

IVUS was performed after a 200 µg dose of intracoronary nitroglycerin using a commercially available imaging system (iMap, Boston Scientific, Natick, MA, USA), an automated motorized pullback system (0.5 mm/s), and the corresponding 40 MHz IVUS catheter (Atlantis SR Pro., Boston Scientific, Natick, MA, USA). After guidewire crossing, the IVUS catheter was carefully advanced 10 mm distal to the culprit lesion and was pulled back automatically to the LMCA ostium. Images were recorded continuously for offline analysis.

### IVUS analysis method

Commercially available software (ImageViewer_05_14_2018_1, Boston Scientific, Corporation/Scimed, Natick, MA) was used. IVUS images of the distal LMCA and its branches were reviewed offline by an experienced observer who was blinded to individual patient data. The standards for the determination of IVUS-RI, IVUS-h-D and IVUS-h-OM on IVUS when withdrawing from LAD were as follows: if a SB could be seen at the entrance of LCX, and the three lumens had blood flow, the presence of RI was confirmed; if a SB was fully incorporated into the LAD before LCX entered, it was considered a h-D; if no SB could be seen within 2 mm before LCX entered, a h-OM was considered to be present.

### Statistical analysis

All statistical analyses were conducted using R (version 3.5.1, https://www.r-project.org/). Continuous variables are shown as the mean ± SD or median (IQR) according to the distribution of the data, and categorical variables are shown as N (%). The *P* values for intergroup differences were calculated using the Kruskal–Wallis test for continuous variables and the chi-squared test or Fisher’s exact test for categorical variables. A *P* value of less than 0.05 was considered to indicate statistical significance.

## Results

### Patient enrollment and characteristics

**Fig. 1:**
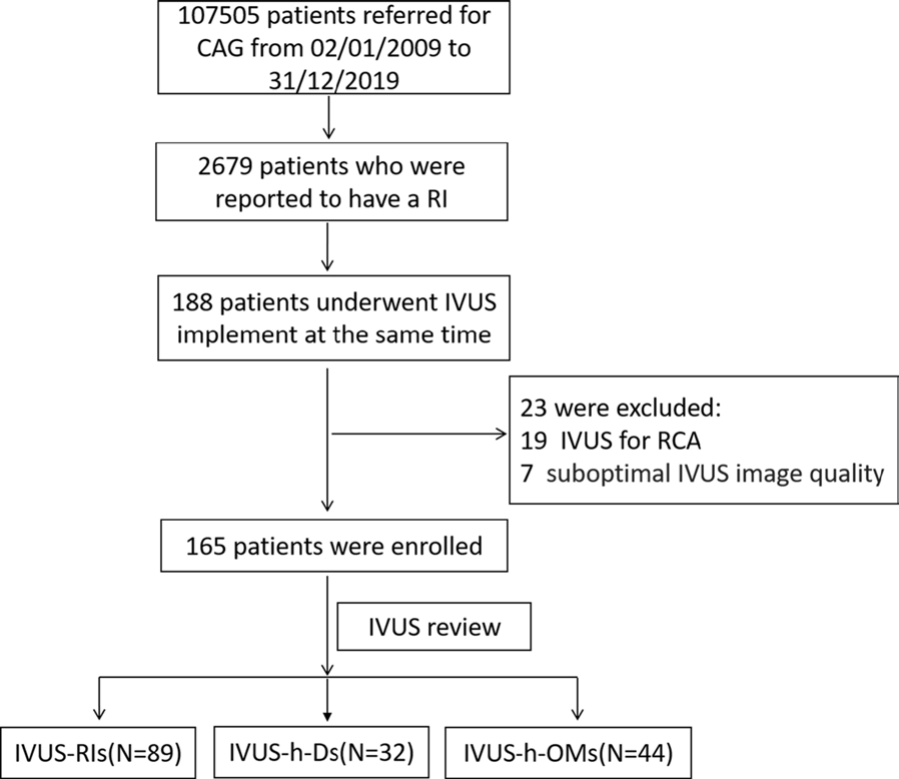
Flowchart of participant enrollment and grouping. The study ultimately included 165 patients. These patients were grouped according to the results of their IVUS reviews, and in every group, only patients who underwent LMCA-LAD crossover stent deployment were included for comparison. RI, ramus intermedius; h-D, high-origin diagonal branch; h-OM, high-origin obtuse marginal artery; LAD, left anterior descending branch; LMCA, left main coronary artery; IVUS, intravascular ultrasonography

Of the 107,505 patients referred for CAG from 02/01/2009 to 31/12/2019, 2679 patients (2.5%) were reported to have an RI, and 188 of those 2679 patients underwent IVUS. After the exclusion of patients with right coronary artery withdrawal (19 patients) or suboptimal IVUS image quality (4 patients), the study ultimately included 165 patients (the mean age was 64.8 ± 10.4 years old, and 16% were female). According to the results of their IVUS review, they were divided into three groups. The flowchart of participant enrollment is shown in Fig. 1. Table 1 summarizes the baseline clinical characteristics and procedural details of the study cohort.

**Table 1:** Baseline characteristics of 165 patients undergoing IVUS analysis

Variable	N (%)
Age, years	64.8 ± 10.4
Female (%)	26 (16)
Current smoker (%)	79 (48)
Disease history	
Hyperlipidemia (%)	14 (8)
Hypertension (%)	64 (39)
Diabetes Mellitus (%)	27 (17)
PCI history (%)	46 (28)
CABG history (%)	3 (2)
Treatment	
Medicine alone (%)	25 (15)
PCI (%)	140 (75)
Trifurcation (%)	93 (66)^a^
Crossover strategy (%)	84 (90)^b^

PCI, percutaneous coronary intervention; CABG, coronary artery bypass graft

^a^Among 140 patients accepted PCI treatment, 93 patients had trifurcation lesion

^b^84 of 93 trifurcation lesion were treated by LMCA-LAD crossover stenting

### IVUS Findings

**Fig. 2:**
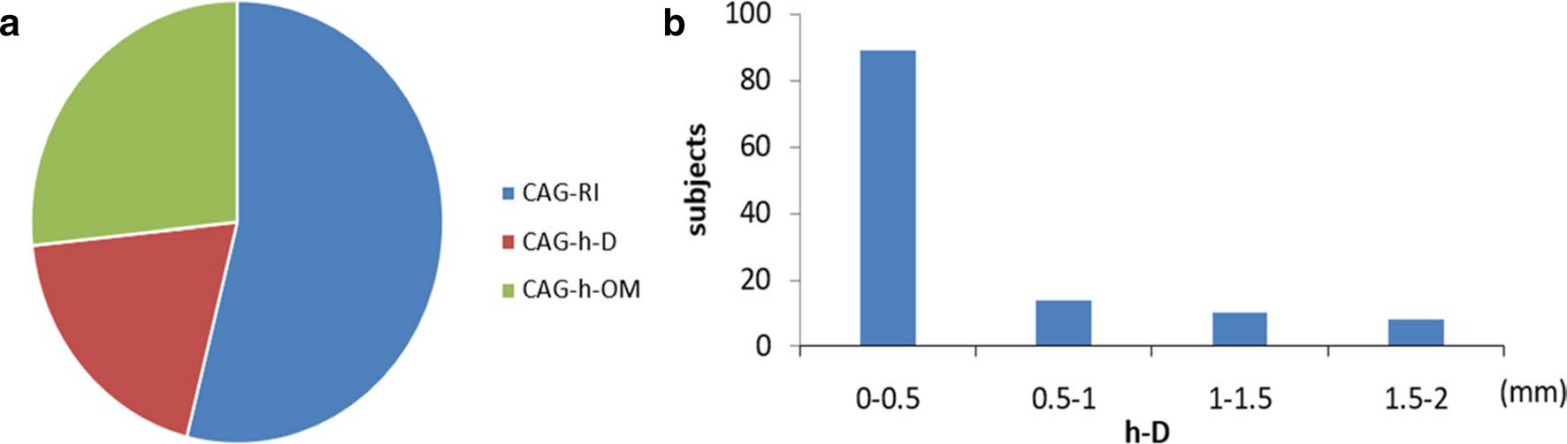
Proportions of IVUS-RI, IVUS-h-D and IVUS-h-OM in IVUS views and the distribution characteristics of IVUS-h-D on IVUS. **a** The pie chart shows that only 54% of CAG-RIs were confirmed by IVUS, and the proportions of IVUS-h-D and IVUS-h-OM were 19% and 27%, respectively. **b** Distance from ostial h-D to the LM furcation carina (mm). RI, ramus intermedius; h-D, high-origin diagonal branch; h-OM, high-origin obtuse marginal artery; IVUS, intravascular ultrasonography; LM, left main

**Fig. 3:**
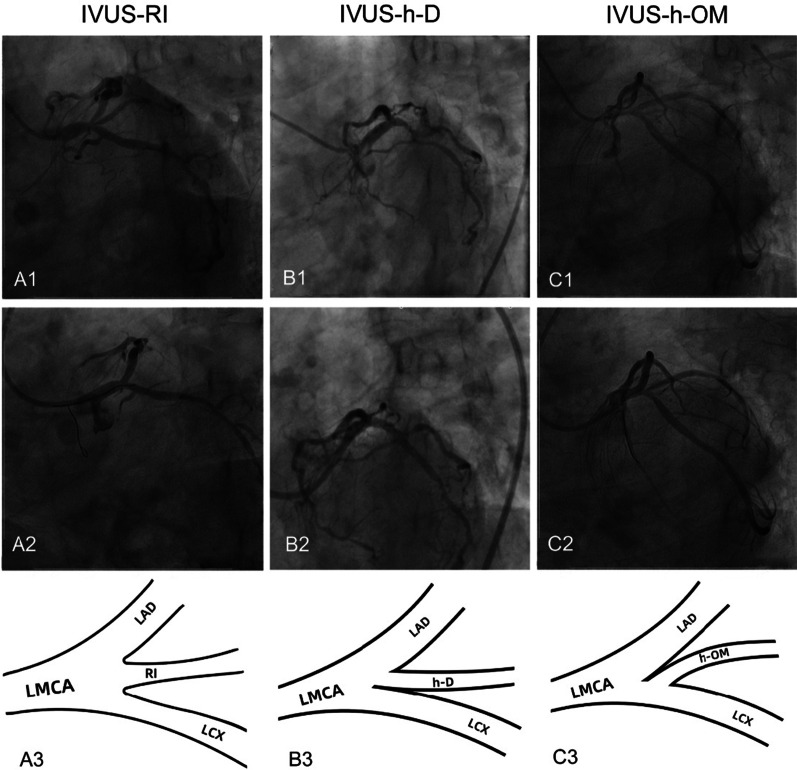
Examples of IVUS-RI, IVUS-h-D and IVUS-h-OM in CAG and their diagrams. Top panel: A left anterior oblique caudal view (“spider” view) revealed a CAG-RI in the distal LM furcation in each group. Second panel: Different outcomes in groups treated with different revascularization strategies; A2: no jailed wire was used in IVUS-RI, and RI occlusion occurred; B2: a jailed wire was used in IVUS-h-D, and no slow flow or lumen reduction occurred; C2: no jailed wire was used in IVUS-h-OM, and no slow flow or lumen reduction occurred. Bottom panel: Diagrams of IVUS-RI, IVUS-h-D and IVUS-h-OM; A3: RI results from the trifurcation point; it can be seen at the entrance of the LCX, and the three lumens have blood flow; B3: IVUS-h-D was fully incorporated into the LAD before the LCX entered; C3: IVUS-h-OM was fully incorporated into the LCX before the LCX entered, but it cannot be seen from the LMCA-LAD IVUS view. RI, ramus intermedius; h-D, high-origin diagonal branch; h-OM, high-origin obtuse marginal artery; LAD, left anterior descending branch; LCX, left circumflex artery; CAG, coronary artery angiography; IVUS, intravascular ultrasonography; LMCA, left main coronary artery

**Fig. 4:**
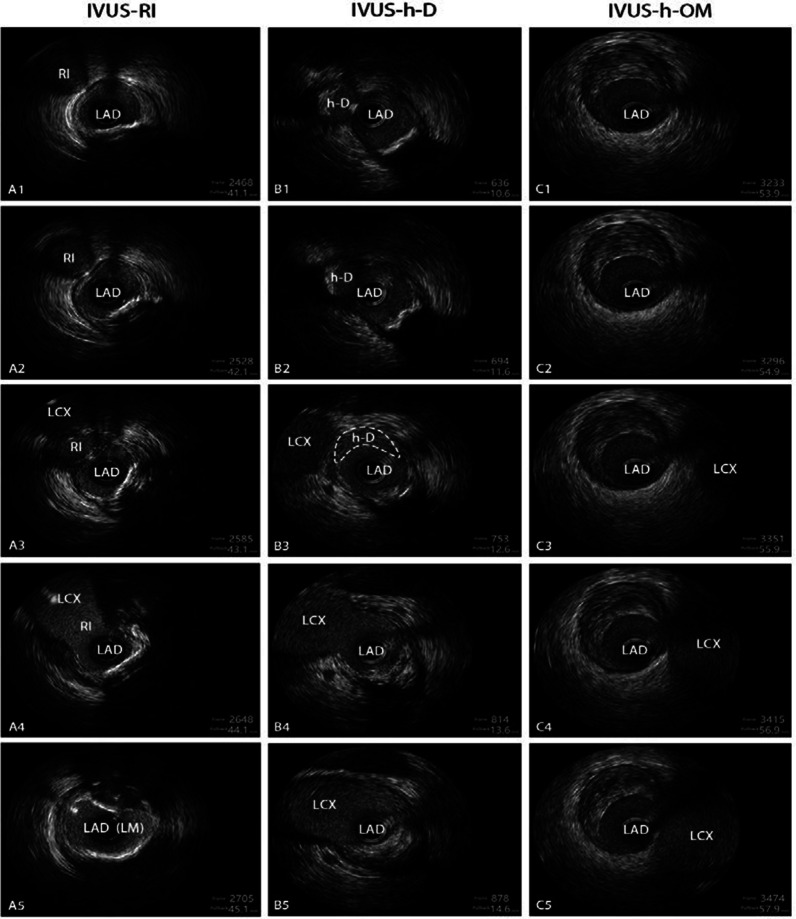
A series of IVUS cross-Sects. (1 mm apart) simulating a pullback from LAD to LMCA for IVUS-RI, IVUS-h-D and IVUS-h-OM. The first column is IVUS-RI, we can see the three lumens have blood flow in A3, the presence of RI is confirmed. The second column is IVUS-h-D, it is fully incorporated into the LAD before LCX entered (B3), an IVUS-h-D is confirmed; The last column is IVUS-h-OM, no SB could be seen within 2 mm before LCX entered, an IVUS-h-OM is considered

IVUS was taken as the gold standard. Twenty IVUS pullbacks were from both the LAD and LCX to LMCA, 145 pullbacks were just from the LAD to LMCA. only 89 patients (54%) were demonstrated to have an RI on IVUS (IVUS-RIs), 32 patients (19%) were identified as having an h-D on IVUS (IVUS-h-Ds) and 44 patients (27%) were considered to have an h-OM on IVUS (IVUS-h-OMs) (Fig. 2a). The distance between the entrance of the IVUS-h-D and the carina of the furcation ranged from 0.5 to 2 mm (Fig. 2b). In some cases, the ostial IVUS-h-D was very close to the carina of the furcation, which may explain why it was easily misjudged as RI on CAG. Figure 3 shows examples of IVUS-RI, IVUS-h-D and IVUS-h-OM in CAG (before and after PCI), and the diagrams of IVUS-RI, IVUS-h-D and IVUS-h-OM are shown in the bottom panel of Fig. 3. Figure 4 illustrates a series of IVUS cross-sections (1 mm apart) simulating a pullback from LAD to LMCA, for IVUS-RI, IVUS-h-D and IVUS-h-OM, respectively.

### The impact of RI on revascularization strategy

In general, one-stent crossover stenting is considered the standard method for most coronary bifurcation lesions. In our study, 90% (84/93) of LMCA furcation lesions were treated with LMCA-LAD crossover stenting. To evaluate the impact of crossover stenting on different angiographically judged RIs (CAG-RIs) and the impact of CAG-RI on the revascularization strategy for LM furcation, we divided the 84 patients who underwent LMCA-LAD one-stent crossover stenting into an IVUS-RI group (N = 48), an IVUS-h-D group (N = 12), and an IVUS-h-OM group (N = 24). The clinical characteristics and procedural details of these 84 patients are shown in Table 2.

**Table 2:** Baseline and operation Characteristics of patients who accepted LMCA-LAD crossover stenting in the three groups

	Total (N = 84)	IVUS-RIs group (N = 48)	IVUS-h-Ds group (N = 12)	IVUS-h-OMs group (N = 24)	*P* value
Age, years	64.1 ± 11.0	64.0 ± 10.9	72.4 ± 8.6	60.2 ± 10.4	0.32
Female (%)	7 (8.3)	3 (6.3)	0 (0)	4 (16.7)	0.17
Current smoker (%)	40 (47.6)	22 (45.8)	5 (41.7)	13 (54.1)	0.72
Diseases history					
Hypertension (%)	33 (39.3)	19 (39.6)	4 (33.3)	10 (41.7)	0.89
Hyperlipidemia (%)	8 (9.5)	5 (10.4)	1 (8.3)	2 (8.3)	0.95
Diabetes mellitus (%)	14 (16.7)	7 (14.5)	2 (16.7)	5 (20.8)	0.8
PCI history (%)	25 (29.8)	14 (29.2)	4 (33.3)	7 (29.2)	0.96
CABG history (%)	2 (2.4)	1 (2.1)	0 (0)	1 (4.2)	0.73
Medina (%)					0.57
0,1,0	46 (54.8)	26 (54.2)	6 (50.0)	14 (58.3)	
0,1,1	13 (15.5)	10 (20.8)	2 (16.7)	1 (4.2)	
1,0,0	6 (7.1)	2 (4.2)	1 (8.3)	2 (8.3)	
1,1,0	15 (17.9)	6 (12.5)	3 (25.0)	6 (25.0)	
1,1,1	5 (6.0)	4 (8.3)	0 (0)	1 (4.2)	
RI, %	30 (0–60)	30 (0–60)	50 (22.5–80)	30 (0–50)	0.33
Plaque burden, %	84.5 (76–90)	88 (78.75–90.5)	83.5 (74–87.25)	78.5 (76–89)	0.05
PCI					
Stent size, mm	3.5 (3.5–3.5)	3.5 (3.0–3.5)	3.5 (3.5–3.5)	3.5 (3.5–3.625)	0.83
Stent balloon pressure, atm	12.0 (12.0–14.0)	12.0 (12.0–14.0)	12.0 (12.0–12.0)	12.0 (12.0–12.0)	0.24
Post balloon size, mm	3.5 (3.5–4)	3.5 (3.5–4.0)	3.63 (3.5–4)	3.75 (3.5–4)	0.63
Post balloon pressure, atm	18.0 (16.0–20.0)	18.0 (15.0–20.0)	20.0 (19.5–20.0)	18.0 (16.0–18.5)	0.12
CAG-RI protection					0.40
Jailed wire, %	46 (54.8)	29 (60.4)	5 (41.7)	12 (50.0)	
Jailed balloon, %	3 (4.8)	2 (2.1)	1 (8.3)	0	
Radiation medium, ml	210 (167.5–300)	220 (180–301.25)	190 (157.5–220)	200 (167.5–302.5)	0.27
Radiation, mGy	900 (780–1502.25)	1005 (795–1575)	795 (630–1062.5)	835 (772.5–1531.75)	0.22
Operation time, min	100 (80–120)	110 (87.5–130)	85 (80–112.5)	90 (80–120)	0.24
Outcome					
Combined outcome*, %	11 (13.1)	7 (14.6)	4 (33.3)	0 (0)	0.02
Rescue balloon inflated, %	7 (8.3)	5 (10.4)	2 (8.3)	0 (0)	0.17

Continuous variables were shown as mean ± SD or median (IQR) according to data distribution, and categorical variables were shown as N (%). The *P* values for inter-group differences were calculated using Kruskal–Wallis test for continuous variables and Chi-squared Test or Fisher exact test for categorical variables

*Severe stenosis of the RI ostium (> 75%) or significant RI flow impairment (TIMI < 3) or both

There were 31 patients (65%) in the IVUS-RI group (29 treated with the jailed wire technique, 2 with the jailed balloon technique), 6 patients (50%) in the IVUS-h-D group (5 jailed wire and 1 jailed balloon procedure), and 12 patients (50%) in the IVUS-h-OM group (all treated with the jailed wire technique) for whom an “RI” protective technique was used. Seven patients (14.6%) in the IVUS-RI group and 4 patients (33.3%) in the IVUS-h-D group had CAG-RI compromise, while no patient in the IVUS-h-OM group had CAG-RI compromise; there was a significant difference among the three groups in this respect (*P* = 0.02). Five of 7 patients in the IVUS-RI group and 2 of 4 patients in the IVUS-h-D group received rescue balloon inflation. There was no significant difference between the two groups regarding the rate of jailed wire/balloon use or the rate of rescue balloon inflation (P > 0.05 for both) (Table 2).

Seven of the 11 patients (63.6%) with CAG-RI compromise, 42 of the 73 patients without CAG-RI compromise underwent the jailed wire/balloon procedure. There was no significant difference about the rate of jailed wire/balloon technique between the two groups of patients. Table 3 summarized the possible factors of RI compromise in the patients with and without RI compromise. Stenosis of RI was higher in patients with RI compromise compared with those without RI compromise (60 [45–65] vs. 30 [0–50] %, P < 0.01). As shown in Table 4, there was a significant association between the stenosis of RI and RI compromise (*P* = 0.049). After additional correction for age and sex, it was still borderline significant (*P* = 0.051), which may be caused by the low sample size. Each 10% increment in RI stenosis increased the risk of RI compromise by 25%.

**Table 3:** Characteristics of patients with and without RI compromise

	RI compromise (N = 11)	No RI compromises (N = 73)
Age, years	67.6 ± 11.5	63.6 ± 10.9
Female, %	0 (0)	7 (9.6)
Stenosis of LAD, %	85.5 ± 13.5	88.7 ± 11.1
Stenosis of RI, %	60 (45–65)	30 (0–50)*
RI jailed wire/balloon, %	7 (63.6)	42 (57.5)
Plaque burden of LAD, %	79.6 ± 12.6	83.1 ± 9.6
Stent diameter, mm	3.45 ± 0.35	3.47 ± 0.37
Stent pressure, atm	11.64 ± 1.5	12.36 ± 1.6
Post balloon diameter, mm	3.59 ± 0.53	3.72 ± 0.5
Post balloon pressure, atm	17.09 ± 4.8	17.86 ± 3.33

Continuous variables were shown as mean ± SD or median (IQR) according to data distribution, and categorical variables were shown as N (%). Asterisk represented a *P* value smaller than 0.05 for inter-group difference in Wilcox rank-sum test

**Table 4:** Multivariate analysis of risk factors for RI compromise

	Odd Ratio	95% CI	*P* value
Age, years	1.05	0.98–1.12	0.20
Female, %	1.00	0.99–1.01	0.99
Stenosis of RI, %	1.26	1.01–1.61	0.05*
RI jailed wire/balloon, %	1.97	0.53–7.50	0.31
Post-balloon pressure, atm	0.91	0.74–1.12	0.38
Plaque burden of LAD, %	1.02	0.62–1.68	0.92

*P = 0.049. After additional correction for age and sex, *P* = 0.051

## Discussion

The current study found that only 54% of angiographically judged RIs were confirmed by IVUS; IVUS-h-OM is rarely occluded during LMCA-LAD crossover stenting. Our findings suggest that preintervention IVUS is necessary to distinguish IVUS-h-OM from IVUS-RI and IVUS-h-D, and the revascularization strategy also needs to be tailored to different types of CAG-RIs.

Typically, LM bifurcates in LAD and LCX. Sometimes, an additional artery, known as RI, arises at the bifurcation of the LM, forming a trifurcation [17, 18]. RI can be identified by CAG; however, standard angiographic projections of CAG are often associated with vessel foreshortening and anatomical overlap [12]. The rate of RI detection was lower on CAG than on CTA. In our study, only 2.5% of patients who underwent CAG were reported to have RIs, lower than the ~ 20% observed by CTA [3, 4]. The shadowgraphic nature of CAG are the varies diameter of RI may explain the low occurrence rate of RI reported by CAG. Furthermore, the interventional cardiologists often pay little attention to the non- target vessel or ignore RI in emergency ACS cases. These are also the reasons why the occurrence rate of RI reported by CAG is low. In contrast to the two-dimensional, shadowgraphic nature of coronary angiography, IVUS is an accurate tomographic technique for anatomical evaluation of the coronary artery [19]. It can accurately distinguish among RI, h-D, and h-OM, helping interventional cardiologists choose stenting and branch protection strategies. In this study of 165 LMCA-LAD IVUS images, we demonstrated that only 54% of CAG-RIs were confirmed as IVUS-RI, 32 CAG-RIs were identified as IVUS-h-D (19%) and 44 CAG-RIs were identified as IVUS-h-OM (27%).

The possible reasons for the lower detection rate of CAG are as follows: (1) RI has a similar course and perfusion region to h-OM or h-D, and it is easily misjudged. (2) Due to the different course of the coronary artery, the distal LM furcation and proximal LAD/LCX of many patients cannot be fully viewed in the conventional left anterior oblique caudal view or right anterior oblique caudal view, which leads to an unclear display of the ostial RI and can cause misjudgment [20]. (3) The conventional projection angle for LMCA furcation in our hospital is LAO 45°/CAU 30°, whereas Kocka et al. [21] reported that optimal fluoroscopic viewing angles for the LMCA bifurcation were LAO 0°/CAU 49° in CTA imaging. This means that a significant proportion of bifurcation views lie outside the practical projection range. (4) Interventional cardiologists do not pay attention to RI, especially when the target lesion is not at the LMCA furcation. (5) Even if an interventional cardiologist wishes to change the fluoroscopic viewing angles to observe LM furcation clearly, not all CTA-defined fluoroscopic viewing angles are practical or achievable with existing C-arm equipment across patients.

PCI procedures for LM bifurcation lesions are still difficult because the jailed wire or jailed balloon technique must be used [9, 10, 22]. In general, one-stent crossover stenting is considered the standard method for most coronary bifurcation lesions [23, 24]. However, it is associated with a risk of SB occlusion after MV stenting [25] because of a combination of carina shift and plaque shift into the SB [26, 27]. Intervention for a trifurcation lesion is more complicated, requiring more wires and various complex interventional techniques [28]. Studies have shown an increase in periprocedural complications (dissection, acute side branch occlusion, periprocedural myocardial infarction) in trifurcation diseases [29, 30]. Among the 84 patients who underwent LMCA-LAD one-stent crossover stenting, 7 patients (14.6%) in the IVUS-RI group, 4 patients (33.3%) in the IVUS-h-D group and no patients (0%) in the IVUS-h-OM group had CAG-RI compromise (narrowing/occlusion) (*P* = 0.02). The probability of compromise is more than twice as high in IVUS-h-D patients as in IVUS-RI patients. However, if the CAG-RI is actual an IVUS-h-OM, the probability of compromise will be lower after LMCA-LAD crossover stenting.

There may be a lot of cofounding factors that influence RI compromise. To discuss the risk of RI compromise, univariate and multivariate analysis were performed. There were no significant differences about the rate of jailed wire/balloon technique, plaque burden of LAD, post-ballooning pressure between patients with and without CAG-RI compromise. However, the stenosis of CAG-RI in patients with RI compromise was higher compared with those without RI compromise. Multivariate analysis showed that the stenosis of RI was an independent risk factor of RI compromise (*P* = 0.049). Each 10% increment in RI stenosis increases the risk of RI compromise by 25%. Therefore, not only the image characteristics of CAG-RI in IVUS, but also the stenosis severity of CAG-RI should be paid attention to.

The jailed wire/balloon technique has been shown to improve the rates of SB reopening in the event of closure [31]. If too many guidewires are used, they will become entangled and make the PCI procedure more difficult. Hence, it is of great value to distinguish IVUS-RI and IVUS-h-D from IVUS-h-OM in LM trifurcation lesions. When crossover stenting is performed in LMCA-LAD for LM furcation lesions with IVUS-RI or IVUS-h-D, a jailed guidewire is needed to protect the IVUS-RI or IVUS-h-D, if the branch is large (> 2 mm), or there is severe stenosis of IVUS-RI or IVUS-h-D, a jailed balloon may be used. For LM furcation lesions with IVUS-h-OM, except for LCX jailed wire procedures, no jailed wire is needed to protect the IVUS-h-OM, which will simplify the trifurcation lesion to a bifurcation lesion and thus reduce the volume of contrast agent, the amount of radiation exposure, and the procedural time. Therefore, in agreement with a recent meta-analysis [32], we suggest that preintervention IVUS should be performed in LM furcation lesions to distinguish among IVUS-RI, IVUS-h-D, and IVUS-h-OM, and the revascularization strategy also needs to be tailored to different types of CAG-RIs.

### Study limitations

The study has inherent limitations owing to its single-center, retrospective design and relatively small sample size, which might introduce selection bias. In addition, the low occurrence rate of RI reported by CAG may be another selection bias. Third, IVUS was performed in LAD alone in most cases, and we could not evaluate the ostial LAD, RI and LCX simultaneously. Last, the plaque load of CAG-RI can also affect the RI blood flow after a one-stent crossover strategy stent implantation [33]. We did not evaluate the plaque burden in RI because of the retrospective design and the IVUS pullbacks were withdrawn from LAD to LMCA. Further prospective investigation is warranted to evaluate RI characteristics and its impact on PCI more accurately.

## Conclusions

In conclusion, the current study illustrated that only 54% of CAG-RIs were confirmed by IVUS, which necessitates preintervention IVUS implementation in LM furcation lesions.

## Data Availability

All data and material used and/or analyzed during the current study are available from the corresponding author on reasonable request.

## Abbreviations

CAG: Coronary artery angiography
CTA: Computed tomography angiography
CRA: Cranial
CAU: Caudal
h-D: High-origin diagonal branch
h-OM: High-origin obtuse marginal artery
IVUS: Intravascular ultrasonography
LAD: Left anterior descending artery
LAO: Left anterior oblique
LCX: Left circumflex artery
LMCA: Left main coronary artery
MV: Main vessel
PCI: Percutaneous coronary intervention
RAO: Right anterior oblique
RI: Ramus intermedius
SB: Side branch
